# Effects of elastase-induced emphysema on muscle and bone in mice

**DOI:** 10.1371/journal.pone.0287541

**Published:** 2023-06-23

**Authors:** Daichi Matsumura, Naoyuki Kawao, Katsumi Okumoto, Takashi Ohira, Yuya Mizukami, Masao Akagi, Hiroshi Kaji

**Affiliations:** 1 Department of Orthopaedic Surgery, Kindai University Faculty of Medicine, Osakasayama, Osaka, Japan; 2 Department of Physiology and Regenerative Medicine, Kindai University Faculty of Medicine, Osakasayama, Osaka, Japan; 3 Life Science Research Institute, Kindai University, Osakasayama, Osaka, Japan; Universitair Kinderziekenhuis Koningin Fabiola: Hopital Universitaire des Enfants Reine Fabiola, BELGIUM

## Abstract

Chronic obstructive pulmonary disease (COPD) causes sarcopenia and osteoporosis. However, the mechanisms underlying muscle and bone loss as well as the interactions between muscle and bone in the COPD state remain unclear. Therefore, we herein investigated the effects of the COPD state on muscle and bone in mice intratracheally administered porcine pancreatic elastase (PPE). The intratracheal administration of PPE to mice significantly reduced trabecular bone mineral density (BMD), trabecular bone volume, trabecular number, cortical BMD and cortical area. It also significantly decreased grip strength, but did not affect muscle mass or the expression of myogenic differentiation-, protein degradation- or autophagy-related genes in the soleus and gastrocnemius muscles. Among the myokines examined, myostatin mRNA levels in the soleus muscles were significantly elevated in mice treated with PPE, and negatively related to grip strength, but not bone parameters, in mice treated with or without 2 U PPE in simple regression analyses. Grip strength positively related to bone parameters in mice treated with or without PPE. In conclusion, we showed that a PPE model of COPD in mice exerts dominant effects on bone rather than skeletal muscles. Increased myostatin expression in the soleus muscles of mice in the COPD state may negatively relate to a reduction in grip strength, but not bone loss.

## Introduction

Chronic obstructive pulmonary disease (COPD) is an inflammatory lung disease characterized by the destruction of alveolar walls [1]. A decrease in quality of life and increased mortality are important in the patients with COPD [1]. COPD has extrapulmonary comorbidities, including osteoporosis and sarcopenia [2]. It has been identified as an independent risk factor for osteoporosis and fractures in the elderly [3]. Sarcopenia is characterized by decreases in skeletal muscle function and mass. The prevalence of sarcopenia was previously shown to be higher in COPD patients than in a healthy elderly population [4]. Nutrition, steroids, inactivity and systemic inflammation may contribute to bone loss and skeletal muscle wasting in COPD patients [5, 6]; however, the mechanisms by which COPD induces osteoporosis and sarcopenia have not yet been elucidated in detail [1, 6, 7].

Based on the clinical relationship between sarcopenia and osteoporosis, interactions between skeletal muscle and bone have been noted [8, 9]. Muscle and bone affect each other in mechanical and endocrine manners. Skeletal muscle releases numerous humoral factors called myokines that exert effects on distant organs, including bone, through the bloodstream. Myostatin, transforming growth factor (TGF)-β, follistatin, irisin, insulin-like growth factor (IGF)-I, fibroblast growth factor (FGF) 2, interleukin (IL)-6, osteoglycin and olfactomedin 1 (OLFM1) are humoral factors that affect bone [10].

Lifestyle-related diseases, such as diabetes, chronic renal failure and COPD, are related to osteoporosis. The levels of irisin, a proteolytic product of fibronectin type III domain-containing 5 (Fndc5), were shown to be lower in patients with type 2 diabetes mellitus [11]. Moreover, irisin has been positively associated with bone mineral density (BMD) and strength in athletes, and inversely associated with osteoporotic fractures in postmenopausal women with osteoporosis [11]. We previously demonstrated that renal failure reduced the expression of irisin in the skeletal muscles of mice, and also that the administration of irisin attenuated cortical bone loss induced by renal failure [12]. However, the effects of COPD on the interactions between muscle and bone through myokines remain unclear.

The elastase-induced emphysema mouse model has been developed as a COPD-related inflammatory model in which the severity of pulmonary emphysema is controlled by the dose of elastase administered and is not affected by smoking, nutrition or body weight loss [13]. We herein investigated the effects of the COPD state on bone and muscle as well as myokines, which affect bone metabolism, using mice intratracheally administered elastase, as previously reported [13].

## Materials and methods

### Ethics statement

All animal experiments were performed in accordance with the guidelines of the National Institutes of Health and the institutional rules for the use and care of laboratory animals at Kindai University. All procedures were approved by the Experimental Animal Welfare Committee of Kindai University (Permit number: KAME-2022-079). All efforts were made to minimize suffering. Mice were euthanized with excess isoflurane.

### Animal experiments

C57BL/6J mice were purchased from CLEA Japan (Tokyo, Japan). Twelve-week-old male mice were divided into two groups: control (n = 8) and 2 U porcine pancreatic elastase (PPE, FUJIFILM Wako Pure Chemical, Tokyo, Japan) (2 U group, n = 7). In another experiment, they were divided into three groups: control (n = 8), 0.1 U PPE (0.1 U group, n = 11), and 0.5 U PPE (0.5 U group, n = 11). After mice were anesthetized with 2% isoflurane, each dose of PPE dissolved into 50 μl saline was administered into their tracheae, as previously described [13]. An equal volume of saline was administered into the trachea for the control group. Eight weeks after the intratracheal administration of PPE, mice were euthanized with excess isoflurane and tissue samples were collected. Food intake was measured every 3 days during experiments. All mice were fed food and water *ad libitum*.

### Quantitative computed tomography (QCT)

A QCT analysis was performed using an X-ray CT system *in vivo* (Latheta LCT-200; Hitachi Aloka Medical, Tokyo, Japan). Mice were scanned 8 weeks after the intratracheal administration of PPE according to the guidelines of the American Society for Bone and Mineral Research [14], as previously described [15, 16]. We started CT scans after mice were anesthetized with 2% isoflurane, and scan images were obtained using the following parameters: 50 kVp tube voltage, 500 μA tube current, 3.6 ms integration time, 48 mm axial field of view, and voxel sizes of 96 × 192 × 1008 μm for analyses of total fat and muscle mass and 48 × 48 × 192 μm for muscle mass in the lower limbs. The region of interest was defined as the whole body for assessments of total fat and muscle mass. Muscle mass and fat mass were analyzed using LaTheta software (version 3.41).

### Micro-computed tomography (μCT)

Mice were scanned 8 weeks after the intratracheal administration of PPE according to the guidelines of the American Society for Bone and Mineral Research [14]. The distal metaphyseal region of the femur was scanned using CosmoScan GXII (Rigaku Corporation, Yamanashi, Japan) with the following parameters: voxel size = 10 × 10 ×10 μm; X-ray voltage = 90 kV; X-ray tube current = 88 μA; exposure time = 4 min. Beam-hardening artifacts were reduced using copper (0.06 mm) and aluminum (0.5 mm) filters. Prior to the analysis of the bone microstructure, raw images were reconstructed by CosmoScan GX ImageAnalysis Software (Rigaku Corporation) with an isotropic voxel size of 5.5 μm. The microstructural parameters of the femur were assessed using the visualization and analysis software, Analyze 14.0 (AnalyzeDirect, Inc., KS, USA). A 1-mm-thick region from 100 μm proximal to the end of the growth plate was used in the trabecular bone analysis, and the following parameters were assessed: trabecular bone volume fraction (BV/TV), trabecular BMD, trabecular number (Tb.N), trabecular thickness (Tb.Th), trabecular separation (Tb.Sp) and connectivity density (Conn.D). A 1-mm-thick region of the mid-diaphysis of the femur was used in the cortical bone analysis, and the following parameters were assessed: cortical BMD, cortical thickness (Ct.Th) and cortical area (Ct.Ar).

### Histological analysis

Eight weeks after the intratracheal administration of PPE, mice were euthanized with excess isoflurane and the lungs and femurs were removed and fixed with 4% paraformaldehyde in 0.1 M phosphate buffer (pH 7.4) at 4 °C for 24 hours.

The lungs were embedded in paraffin. Four-micrometer-thick sections of the lungs were obtained and stained with hematoxylin/eosin. Hematoxylin/eosin-stained sections were photographed under a microscope (BZ-X810; Keyence, Osaka, Japan). To measure the mean linear intercept (MLI), 10 fields were randomly acquired at 20× magnification, and MLI was measured using ImageJ with a plug-in [17].

Femurs were demineralized in 22.5% formic acid and 340 mM sodium citrate solution for 24 hours and then embedded in paraffin. Five-micrometer-thick sections of the femur were obtained. Immunostaining for alkaline phosphatase (ALP) was performed, as previously described [18]. Femur sections were incubated with an anti-ALP antibody at a dilution of 1:200 followed by a horseradish peroxidase-conjugated secondary antibody. Positive signals were visualized using the tyramide signal amplification system (PerkinElmer, Waltham, MS, USA). Sections were counterstained with 4’,6-diamidino-2-phenylindole and photographed under a fluorescence microscope (BZ-810, Keyence). The number of ALP-positive cells on the surface of trabecular bone was measured in a 1-mm-thick region from 100 μm proximal to the end of the growth plate using ImageJ [18].

Femur sections were stained with tartrate-resistant acid phosphatase (TRAP) using a TRAP staining kit (FUJIFILM Wako Pure Chemical), and counterstained with hematoxylin, as previously described [19]. The number of TRAP-positive multinucleated cells (MNCs), which have more than three nuclei, on the surface of trabecular bone was measured.

### Measurement of grip strength

Eight weeks after the administration of PPE, the grip strength of the limbs was measured using a pull bar attached to a grip strength meter (1027SM, Columbus Instruments, Columbus, OH, USA), as previously described [12]. Mice grasped a pull bar attachment by the limbs. They were then pulled continuously at a rate of approximately 2 cm/sec. This test was performed 5 times and the results obtained represented the average of each mouse.

### Real-time polymerase chain reaction (PCR)

Total RNA from mouse tissues was isolated using an RNeasy Mini Kit (Qiagen, Hilden, Germany), as previously described [12]. A High-Capacity cDNA Reverse Transcription Kit (Applied Biosystems, Foster city, CA, USA) was used for the reverse transcription reaction, and the resulting cDNA was subjected to real-time PCR using an ABI StepOne Real-Time PCR System (Applied Biosystems) with Fast SYBR Green Master Mix (Applied Biosystems). PCR primer sets are shown in S1 Table. The specific mRNA amplification of the target was assessed as the Ct value, which was followed by normalization with 18S ribosomal RNA levels.

### Blood chemistry

Serum tumor necrosis factor (TNF)-α levels were measured using an enzyme-linked immunosorbent assay kit for mouse TNF-α (R&D Systems, Minneapolis, MN, USA, Cat. No. MTA00B).

### Statistical analysis

Data are expressed as the mean ± the standard error of the mean (SEM). The significance of differences was evaluated using the Mann-Whitney *U* test for comparisons of 2 groups. A one-way analysis of variance followed by Dunnett’s test was performed for multiple comparisons. A simple regression analysis was conducted using Pearson’s test. The significance of differences was set at *p* <0.05. All statistical analyses were performed using GraphPad PRISM 7.00 software (GraphPad Software, La Jolla, CA, USA).

## Results

### Effects of the intratracheal administration of PPE on bone parameters in mice

**Fig 1:**
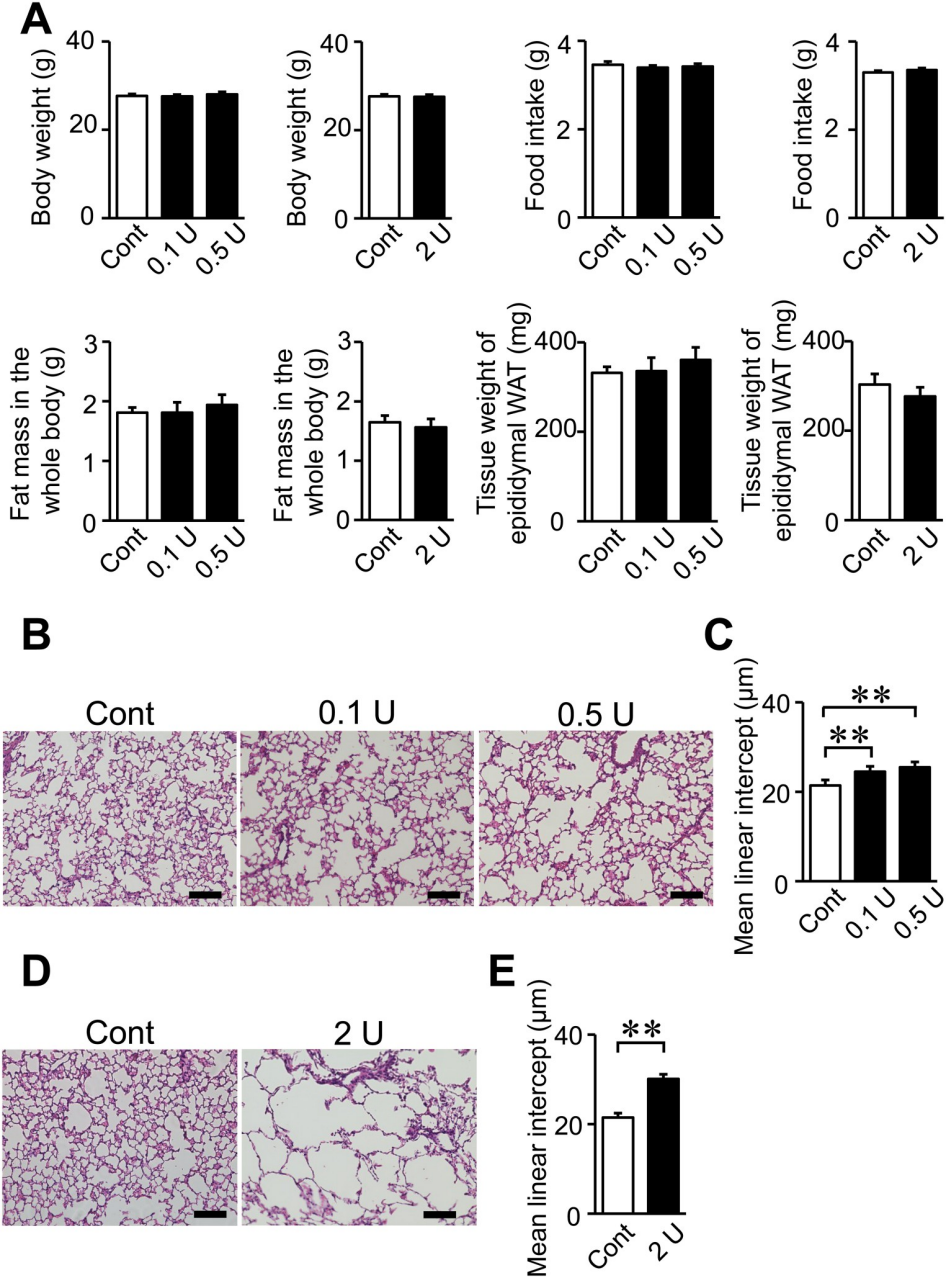
Effects of the intratracheal administration of PPE in mice. (A) Body weight of and food intake by control mice and mice treated with PPE. Body weight was measured 8 weeks after the intratracheal administration of saline or PPE (0.1 to 2 U). Food intake was measured for 3 days on days 54 to 56 after the intratracheal administration of saline or PPE and shown as a representative of average daily food intake. Fat mass in the whole body was assessed by QCT 8 weeks after the intratracheal administration of saline or PPE. The tissue weight of epididymal WAT was measured 8 weeks after the intratracheal administration of saline or PPE. (B, D) Representative images of lung sections stained with hematoxylin/eosin 8 weeks after the intratracheal administration of saline or PPE. Scale bars indicate 100 μm. (C, E) Mean linear intercepts of hematoxylin/eosin-stained lung sections were measured 8 weeks after the intratracheal administration of saline or PPE. Data represent the mean ± SEM. n = 8 (Control), 11 (0.1 and 0.5 U) and 7 (2 U) mice (A, C, E). **p <0.01 and *p <0.05. Cont; Control.

**Fig 2:**
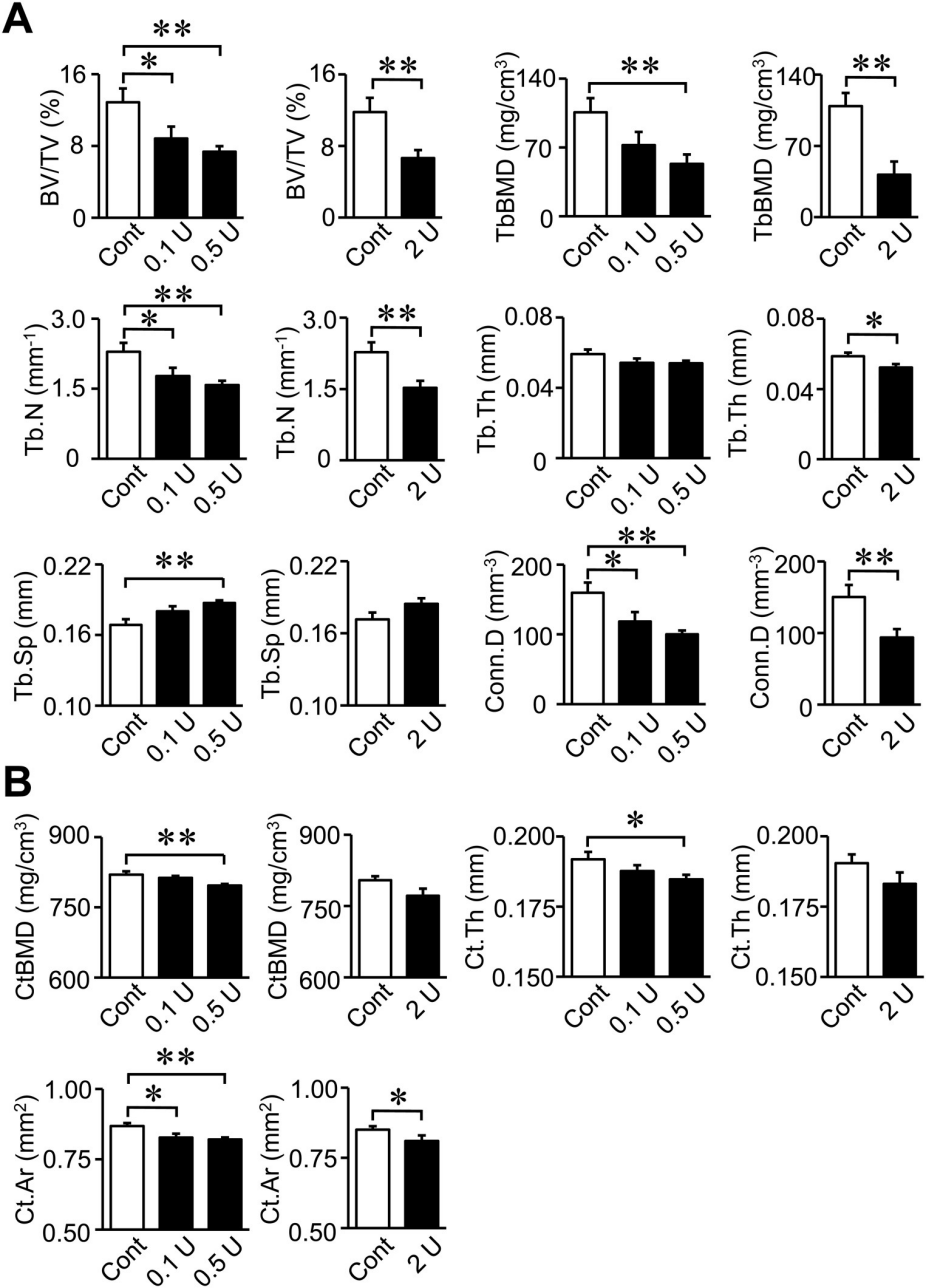
Effects of the intratracheal administration of PPE on bone microstructural parameters measured with μCT. (A) BV/TV, trabecular BMD (TbBMD), trabecular number (Tb.N), trabecular thickness (Tb.Th), trabecular separation (Tb.Sp) and connectivity density (Conn.D) at the distal femurs of mice were assessed by μCT 8 weeks after the intratracheal administration of saline or PPE (0.1 to 2 U). (B) Cortical BMD (CtBMD), cortical thickness (Ct.Th) and cortical area (Ct.Ar) at the femurs of mice were assessed by μCT 8 weeks after the intratracheal administration of saline or PPE. n = 8 (Control), 11 (0.1 and 0.5 U) and 7 (2 U) mice. Data represent the mean ± SEM. **p <0.01 and *p <0.05.

The intratracheal administration of PPE did not affect body weight, food intake, fat mass in the whole body or the tissue weight of epididymal white adipose tissue (WAT) in mice (Fig 1A). The administration of 0.1, 0.5 and 2 U PPE significantly increased MLI in mice, which indicated the successful induction of emphysema (Fig 1B–1E). Trabecular BV/TV, trabecular BMD, Tb.N, Tb.Th and Conn.D were significantly lower in mice treated with 0.5 and 2 U PPE than in control mice (Fig 2A). Moreover, trabecular BV/TV, Tb.N and Conn.D were significantly lower in mice treated with 0.1 U PPE than in control mice. Tb.Sp was significantly higher in mice treated with 0.5 U PPE than in control mice (Fig 2A). Cortical BMD and Ct.Th were significantly lower in mice treated with 0.5 U PPE than in control mice, while Ct.Ar was significantly lower in mice treated with 0.1, 0.5 and 2 U PPE than in control mice (Fig 2B). The number of ALP-positive cells on the surface of trabecular bone was significantly lower in mice treated with 2 U PPE than in control mice, while no significant difference was observed in the number of TRAP-positive MNCs between control mice and mice treated with 2 U PPE (S1 Fig).

### Effects of the intratracheal administration of PPE on the expression of myogenic differentiation-, muscle protein degradation- and autophagy-related genes in mice

**Fig 3:**
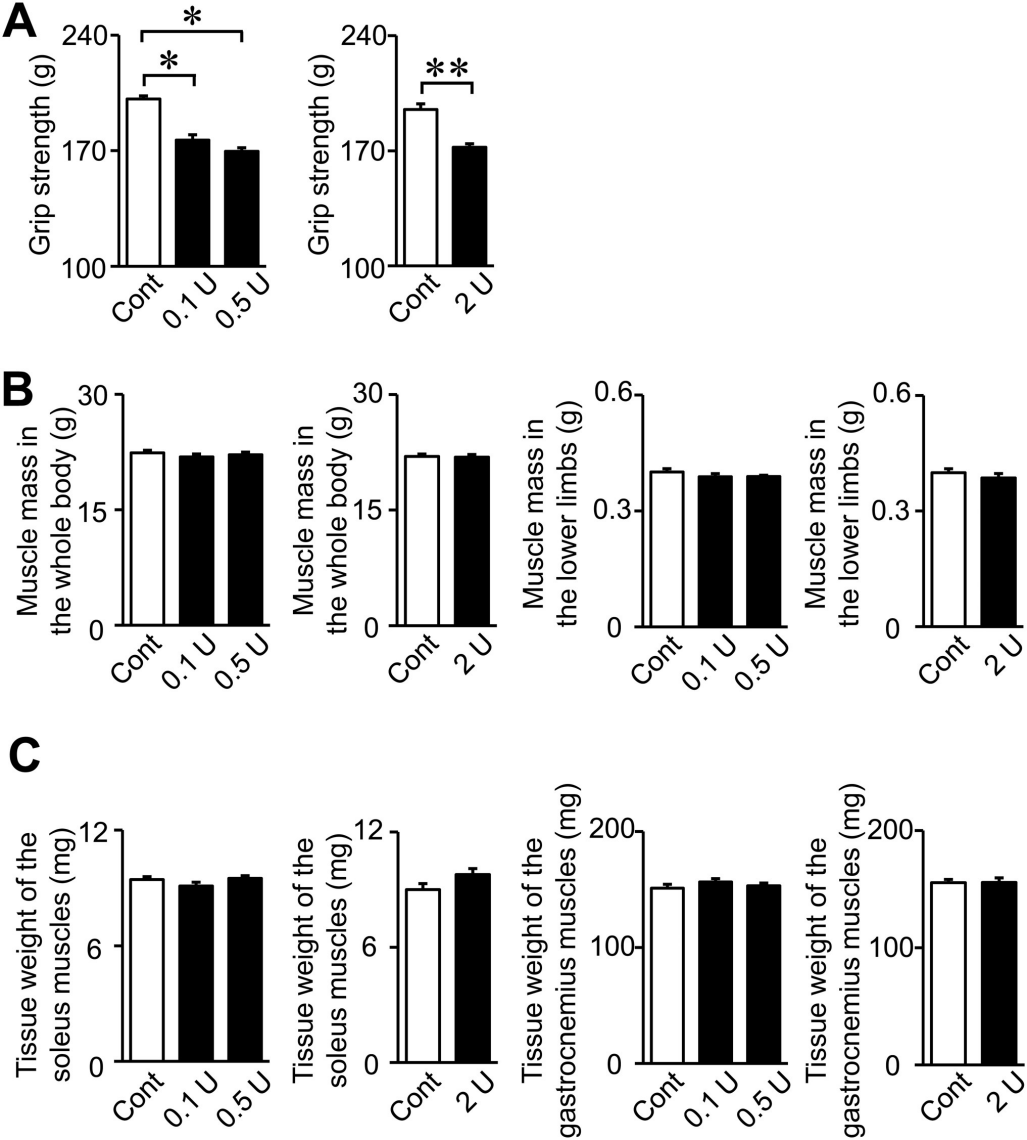
Effects of the intratracheal administration of PPE on muscle strength and muscle mass. (A) The grip strength of the four limbs in mice was measured by a grip strength meter 8 weeks after the intratracheal administration of saline or PPE (0.1 to 2 U). (B) Muscle mass in the whole body and in the lower limbs was assessed by QCT 8 weeks after the intratracheal administration of saline or PPE. (C) The tissue weights of the soleus or gastrocnemius muscles were measured in mice 8 weeks after the intratracheal administration of saline or PPE (2 U). Data represent the mean ± SEM. n = 8 (Control), 11 (0.1 and 0.5 U) and 7 (2 U) mice. **p <0.01 and *p <0.05.

**Fig 4:**
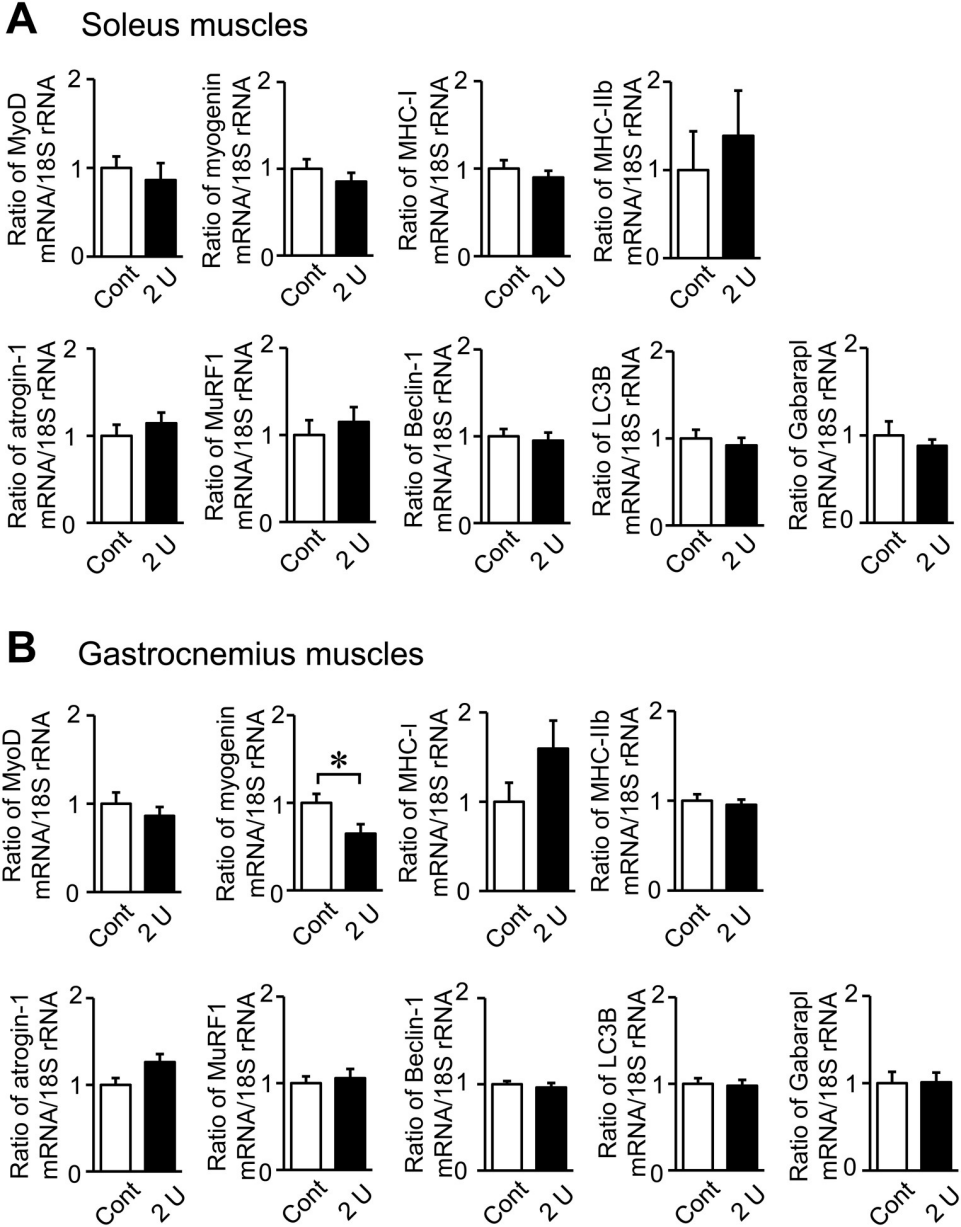
Effects of the intratracheal administration of PPE on muscle-related parameters in mice. Total RNA was extracted from the soleus (A) and gastrocnemius (B) muscles of mice 8 weeks after the intratracheal administration of saline or PPE (2U). A real-time PCR analysis of MyoD, myogenin, MHC-I, MHC-IIb, atrogin-1, MuRF1, Beclin-1, LC3B, Gabarapl and 18S rRNA was performed. n = 8 (Control), 11 (0.1 and 0.5 U) and 7 (2 U) mice. Data are expressed as relative values to 18S rRNA levels and represent the mean ± SEM. *p < 0.05.

The intratracheal administration of 2 U PPE significantly decreased grip strength in mice, but did not affect muscle mass in the whole body and lower limbs or the tissue weights of the soleus and gastrocnemius muscles (Fig 3A–3C). Moreover, the administration of PPE did not affect the mRNA levels of myogenic genes, such as MyoD and myosin heavy chain (MHC) types I and IIb, in the soleus or gastrocnemius muscles (Fig 4A and 4B). The administration of PPE significantly reduced myogenin mRNA levels in the gastrocnemius muscles, but not the soleus muscles (Fig 4A and 4B). No significant differences were observed in the mRNA levels of muscle protein degradation-related E3 ubiquitin ligases, such as atrogin-1 and MuRF1, or autophagy-related genes, including Beclin-1, LC3B and Gabarapl in the soleus or gastrocnemius muscles between control mice and mice treated with 2 U PPE (Fig 4A and 4B).

### Effects of the intratracheal administration of PPE on myokine expression in skeletal muscles of mice

**Fig 5:**
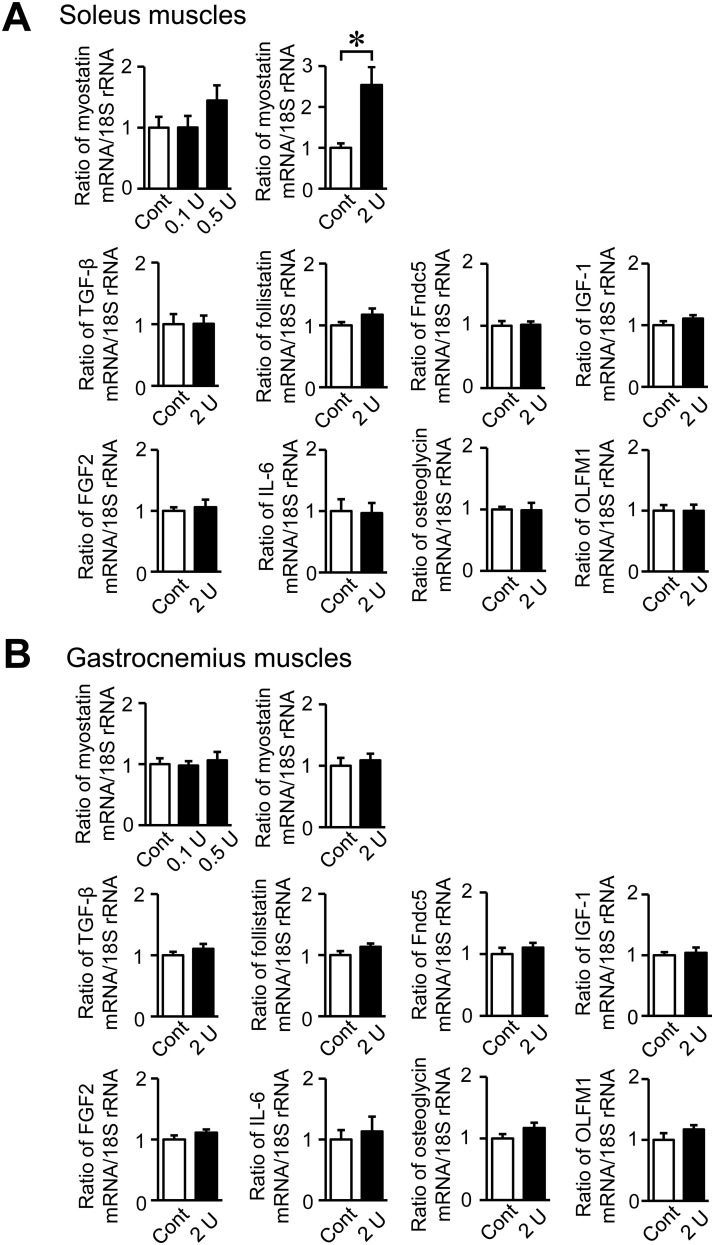
Effects of the intratracheal administration of PPE on myokines in muscles. Total RNA was extracted from the soleus (A) and gastrocnemius (B) muscles of mice 8 weeks after the intratracheal administration of saline or PPE (2 U). A real-time PCR analysis of myostatin, TGF-β, follistatin, Fndc5, IGF-1, FGF2, IL-6, osteoglycin, OLFM1 and 18S rRNA was performed. Data are expressed as relative values to 18S rRNA levels and represent the mean ± SEM. n = 8 (Control), 11 (0.1 and 0.5 U) and 7 (2 U) mice. *p <0.05.

The intratracheal administration of 2 U PPE significantly increased myostatin mRNA levels in the soleus muscles, but not gastrocnemius muscles (Fig 5A and 5B). On the other hand, myostatin mRNA levels in the soleus and gastrocnemius muscles did not significantly differ between control mice and mice treated with 0.1 or 0.5 U PPE (Fig 5A and 5B). The intratracheal administration of PPE did not affect the mRNA levels of TGF-β, follistatin, Fndc5, IGF-1, FGF2, IL-6, osteoglycin or OLFM1 in the soleus and gastrocnemius muscles (Fig 5A and 5B).

### Relationships between the expression of myostatin in the soleus muscles and bone or muscle parameters in mice

We examined the relationships between myostatin expression in the soleus muscles and bone or muscle parameters using simple regression analyses. Myostatin mRNA levels in the soleus muscles negatively correlated with grip strength in control mice and mice treated with 2 U PPE (Table 1). On the other hand, no correlations were observed between myostatin mRNA levels in the soleus muscles and bone parameters or muscle mass (Table 1).

**Table 1:** Relationships between myostatin mRNA levels in soleus muscles and bone or muscle parameters in mice.

	Myostatin mRNA level (SL)	
	r	P
BV/TV	-0.329	0.296
TbBMD	-0.360	0.251
Tb.N	-0.335	0.287
Tb.Th	-0.203	0.526
CtBMD	-0.091	0.777
Ct.Ar	0.025	0.940
Grip strength	-0.651	0.012*
Muscle mass in the lower limbs	-0.204	0.484

A simple regression analysis was performed on myostatin mRNA levels in the soleus muscles and BV/TV, trabecular BMD (TbBMD), Tb.N, Tb.Th, cortical BMD (CtBMD), Ct.Ar, grip strength and muscle mass in the lower limbs of control mice and mice treated with 2 U PPE.

### Relationships between grip strength and bone or muscle parameters in mice

We examined the relationships between grip strength and bone or muscle parameters in mice using simple regression analyses. Grip strength positively correlated with BV/TV, trabecular BMD, Tb.N, cortical BMD and Ct.Ar, but not Tb.Th, in control mice and mice treated with 0.1, 0.5 and 2 U PPE (Table 2). Moreover, grip strength positively correlated with muscle mass in the lower limbs of mice (Table 2).

**Table 2:** Relationships between grip strength and bone or muscle parameters in mice.

	Grip strength	
	r	P
BV/TV	0.364	0.016*
TbBMD	0.390	0.010*
Tb.N	0.378	0.012*
Tb.Th	0.189	0.225
CtBMD	0.311	0.043*
Ct.Ar	0.399	0.008*
Muscle mass in the lower limbs	0.358	0.014*

A simple regression analysis was performed on grip strength and BV/TV, trabecular BMD (TbBMD), Tb.N, Tb.Th, cortical BMD (CtBMD), Ct.Ar and muscle mass in the lower limbs of control mice and mice treated with 0.1, 0.5 and 2 U PPE.

### Effects of the intratracheal administration of PPE on TNF-α in mice

**Fig 6:**
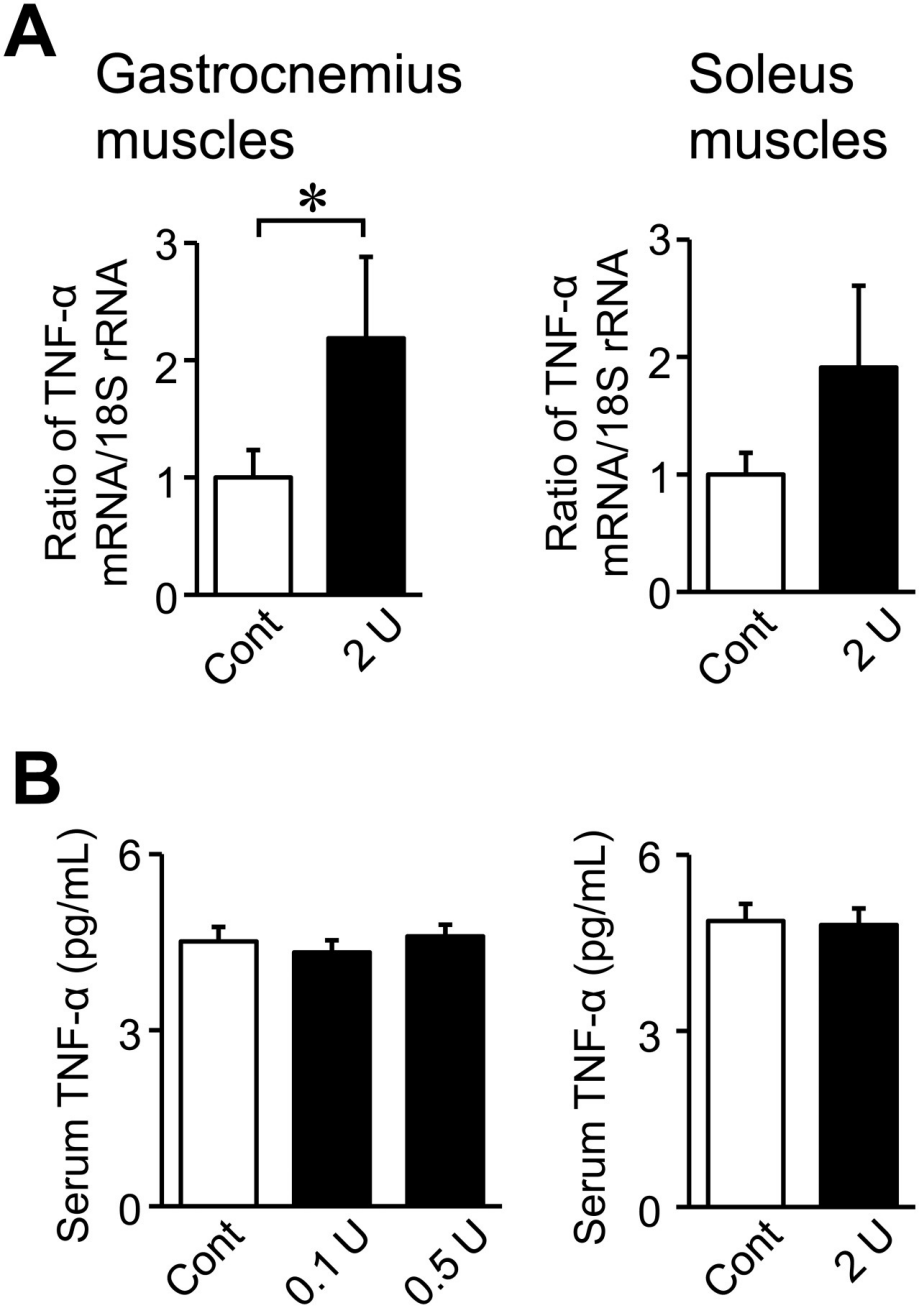
Effects of the intratracheal administration of PPE on TNF-α. (A) Total RNA was extracted from the gastrocnemius and soleus muscles of mice 8 weeks after the intratracheal administration of saline or PPE (0.1 to 2 U). A real-time PCR analysis of TNF-α and 18S rRNA was performed. Data are expressed as relative values to 18S rRNA levels. (B) Serum samples were collected from mice 8 weeks after the intratracheal administration of saline or PPE (0.1 to 2 U). Serum TNF-α levels were analyzed. Data represent the mean ± SEM. n = 8 (Control), 11 (0.1 and 0.5 U) and 7 (2 U) mice. *p <0.05.

We investigated the effects of the administration of PPE on TNF-α expression in mice. The intratracheal administration of 2 U PPE significantly increased TNF-α mRNA levels in the gastrocnemius muscles, but not the soleus muscles (Fig 6A). PPE did not affect serum TNF-α levels in mice (Fig 6B).

## Discussion

In the present study, we showed that the intratracheal administration of PPE reduced trabecular bone volume and microstructural parameters as well as cortical bone parameters in the femurs of mice. On the other hand, it decreased grip strength and increased the expression of myostatin in the soleus muscles of mice. Myostatin expression in the soleus muscles negatively correlated with grip strength, but not bone parameters.

COPD negatively affects bone and muscle and induces osteoporosis and sarcopenia [20]. Skeletal muscle dysfunction is a common systemic manifestation in patients with COPD [4]. In a previous clinical study, trabecular bone volume, trabecular number, trabecular thickness and connectivity density were lower, and trabecular separation and cortical porosity were higher in women with COPD [21]. In the present study, BV/TV, trabecular BMD, trabecular number, trabecular thickness, connectivity density, cortical BMD, cortical thickness and cortical area were lower in mice treated with any dose of PPE than in control mice, and trabecular separation was higher. These results are consistent with previous findings showing trabecular bone loss in similar 24-week-old male elastase-induced emphysema model mice [13]. The effects of the COPD state on bone parameters seemed to be more potent in trabecular bone than in cortical bone. Humoral factors and mechanical stresses often influence trabecular and cortical bone, respectively. We therefore speculated that some humoral factors, such as myokines and inflammatory factors, may be related to the effects of the COPD state on bone than mechanical factors.

We showed that the administration of PPE decreased grip strength and myogenin expression in the gastrocnemius muscles of mice, but did not affect the tissue weights of the soleus and gastrocnemius muscles, muscle mass, or the expression of myogenic differentiation-, muscle protein degradation- and autophagy-related genes in mice. These results suggest that the COPD state dominantly induces bone loss compared to muscle wasting in mice. However, Tsukamoto et al. reported that the COPD state in mice induced the atrophy of type I muscle fibers, but not type II muscle fibers [13]. In that study, even 0.025 U PPE significantly reduced the tissue weight of the soleus muscles, but not the gastrocnemius muscles. This discrepancy may be partly attributed to the age of mice used or the administration method of PPE, while the extent of pulmonary emphysema assessed by a histological analysis of mice seemed to be consistent with that in the present study. Collectively, the present results on the effects of the COPD state on bone and muscle strongly indicate that the COPD state dominantly induces bone loss than muscle wasting in mice. We speculate that factors such as nutrition rather than lung lesions may induce skeletal muscle wasting in the COPD state.

Weakened grip strength, weight loss and muscle loss are common in patients with COPD [22, 23]. Wang et al. reported that the decline in muscle quality was markedly larger than the loss of muscle mass [23]. In the present study, the intratracheal administration of PPE decreased grip strength, but not muscle mass, in mice, which is consistent with previous findings [23]. Therefore, these findings suggest that the COPD state dominantly influences muscle strength and function than muscle mass in mice.

Myostatin belongs to the TGF-β superfamily and is considered to exert negative effects on skeletal muscle [10]. Previous studies reported that myostatin null mice exhibited muscle hypertrophy [24, 25]. Myostatin inhibits the proliferation and differentiation of myoblasts and promotes muscle atrophy [10, 26, 27]. Plant et al. demonstrated that the expression of myostatin was significantly increased in the vastus lateralis muscle of patients with COPD [28]. In the present study, myostatin mRNA levels were significantly elevated in the soleus muscles of mice with PPE. Moreover, myostatin mRNA levels in the soleus muscles negatively correlated with grip strength in simple regression analyses of the mice used in experiments. Therefore, myostatin may be partly associated with diminished muscle strength in COPD.

Myostatin is a well-known myokine that affects bone metabolism [10, 29]. Previous studies showed that myostatin suppressed osteoblast differentiation and promoted RANKL-induced osteoclast formation, causing bone loss through a decrease in bone formation and increase in bone resorption [10, 30, 31]. However, we showed that myostatin mRNA levels in the soleus muscles did not correlate with bone parameters in control mice or mice treated with PPE. These results were compatible with a previous study showing that a myostatin inhibitor (propeptide-Fc) increased muscle mass and muscle fiber size in aged mice, but did not affect bone density or strength [32]. These findings and the present results suggest that myostatin does not contribute to COPD-induced bone loss as a myokine linking muscle to bone in mice.

There are numerous findings to support the relationships between grip strength and bone parameters. Luo et al. revealed that an increase in grip strength was associated with higher BMD at the femoral neck and lumbar spine in men and women [33]. Lin et al. reported that hand grip strength was positively related to bone density and the bone microarchitecture, and also that a decrease in grip strength was a significant predictor of osteoporosis [34]. In the present study, PPE significantly decreased grip strength in mice. Moreover, grip strength positively correlated with BV/TV, trabecular BMD, trabecular number, cortical BMD and cortical area in the mice used in our experiments. These results suggest that a weakened grip strength may partly induce bone loss in elastase-induced COPD mice. Decreased physical activity increases the risk of sarcopenia and osteoporosis in patients with COPD [20]. Previous studies showed that mechanical unloading, such as long-term bed rest and microgravity, reduced both muscle strength and mass, which preceded bone loss [35, 36]. The present study suggested a possibility that a decrease in grip strength may be related to bone loss in the COPD state in mice. Therefore, decreased muscle strength may be a predictor of future bone loss in COPD patients. Since the present results suggested that a PPE model of COPD in mice dominantly induces bone loss than muscle wasting in mice, the COPD state may directly affect bone tissues through various mechanisms, including nutrition, oxidative stress, hypoxia and inflammation, as well as the effects *via* skeletal muscles in mice. Further clinical studies are needed to clarify these issues.

Inflammation is considered to be a cause of sarcopenia. Tuttle et al. reported that higher levels of circulating inflammatory markers correlated with lower skeletal muscle strength and muscle mass [37]. In the present study, the administration of PPE to mice did not affect serum TNF-α levels, but increased TNF-α mRNA levels in the gastrocnemius muscles. These results suggest that local inflammation in muscle tissues, but not systemic inflammation, may partly contribute to the decrease observed in grip strength in elastase-induced COPD mice.

There is the limitation in this study. The PPE model of COPD in mice used in the present study may not represent the real clinical pathophysiology of COPD in humans. Smoking exposure models have been used as a COPD animal model, in which muscle wasting is milder than in human COPD, and the time course of emphysema may differ between the PPE model of COPD in mice and human COPD. However, there are several merits of the PPE model of COPD in mice. Namely, the PPE model exhibits similar characteristics to patients with COPD and smoking animal models, and is affected less by smoking, nutrition and body weight loss. Moreover, the extent of emphysema in the PPE model may be regulated by the administration of different concentrations of elastase [38].

In conclusion, the present study revealed that the administration of PPE induced bone loss and bone microstructural changes in mice. We showed that negative effects by a PPE model of COPD in mice on bone are dominant rather than those effects on the skeletal muscles. The administration of PPE increased myostatin expression in the soleus muscles of mice, suggesting that COPD state-induced myostatin expression may be related to a reduction in grip strength, but not bone loss, in mice.

## Supporting information

S1 Table Primers used for real-time PCR experiments. MHC, myosin heavy chain; Fndc5, fibronectin type III domain-containing 5; IGF-1, insulin-like growth factor-1; FGF2, fibroblast growth factor 2; TGF-β, transforming growth factor-β; IL-6, interleukin-6; OLFM1, Olfactomedin 1.
(DOCX)

S1 Fig Effects of the intratracheal administration of PPE on ALP-positive cells and TRAP-positive multinucleated cells (MNCs) in the distal femur. (A) Immunohistochemistry for ALP was performed on the femurs of mice 8 weeks after the intratracheal administration of saline or 2 U PPE. The number of ALP-positive cells on the bone surface of femoral trabecular bone was counted. (B) TRAP staining was performed on the femurs of mice 8 weeks after the intratracheal administration of saline or 2 U PPE. The number of TRAP-positive MNCs on the bone surface of femoral trabecular bone was counted. Data represent the mean ± SEM. n = 8 (Control) and 5 (2 U) mice. *p <0.05.
(TIF)
