# Supplementary material for: Effects of elastase-induced emphysema on muscle and bone in mice
Source: PLoS One. 2023 Jun 23;18(6):e0287541. doi: 10.1371/journal.pone.0287541 (PMC10289373; doi:10.1371/journal.pone.0287541)
Supplement: S1 Table — MHC, myosin heavy chain; Fndc5, fibronectin type III domain-containing 5; IGF-1, insulin-like growth factor-1; FGF2, fibroblast growth factor 2; TGF-β, transforming growth factor-β; IL-6, interleukin-6; OLFM1, Olfactomedin 1. (DOCX) [file pone.0287541.s001.docx]

**S1 Table.** Primers used for real-time PCR experiments.

| Gene |  | Primer sequence |
| --- | --- | --- |
| MyoD  Myogenin  MHC-I  MHC-IIb  Atrogin-1  MuRF1  Beclin-1 | Forward  Reverse  Forward  Reverse  Forward  Reverse  Forward  Reverse  Forward  Reverse  Forward  Reverse  Forward  Reverse | 5’-AGCACTACAGTGGCGACTCAG-3’  5’-AGGCGGTGTCGTAGCCATTC-3’  5’-GCTGCCTAAAGTGGAGATCCT-3’  5’-GCGCTGTGGGAGTTGCAT-3’  5’-GCCAACTATGCTGGAGCTGATGCCC-3’  5’-GGTGCGTGGAGCGCAAGTTTGTCATAAG-3’  5’-CGAAGGCGGAGCTACGGTCA-3’  5’-CGGCAGCCACTTGTAGGGGT-3’  5’-GTCGCAGCCAAGAAGAGAAAGA-3’  5’-TGCTATCAGCTCCAACAGCCTT-3’  5’-TAACTGCATCTCCATGCTGGTG-3’  5’-TGGCGTAGAGGGTGTCAAACTT-3’  5’-TGAAATCAATGCTGCCTGGG-3’  5’-CCAGAACAGTATAACGGCAACTCC-3’ |
| LC3B | Forward  Reverse | 5’-CTGGTGAATGGGCACAGCATG-3’  5’-CGTCCGCTGGTAACATCCCTT-3’ |
| Gabalapl | Forward  Reverse | 5’-CATCGTGGAGAAGGCTCCTA-3’  5’-ATACAGCTGGCCCATGGTAG-3’ |
| Myostatin  TGF-β  Follistatin  Fndc5 | Forward  Reverse  Forward  Reverse  Forward  Reverse  Forward | 5’-CTGTAACCTTCCCAGGACCA-3’  5’-TCTTTTGGGTGCGATAATCC-3’  5’-GCAACAATTCCTGGCGTTACC-3’  5’-CGCTGAATCGAAAGCCCTGTA-3’  5’-AGAGGAAATGTCTGCTTCCG-3’  5’-CACCTCTCTTCAGTCTCCTG-3’  5’-TCATTGTTGTGGTCCTCTTC-3’ |
|  | Reverse | 5’-GCTCGTTGTCCTTGATGATA-3’ |
| IGF-1 | Forward | 5’-CAAGCCCACAGGCTATGGC-3’ |
|  | Reverse | 5’-TCTGAGTCTTGGGCATGTCAG -3’ |
| FGF2 | Forward | 5’-GCGACCCACACGTCAAACTA-3’ |
|  | Reverse | 5’-CCGTCCATCTTCCTTCATAGC-3’ |
| IL-6  Osteoglycin  OLFM1 | Forward  Reverse  Forward  Reverse  Forward | 5’-GTTCTCTGGGAAATCGTGGA-3’  5’-GGAAATTCGGGGTAGGAAGGA-3’  5’-AATGATGAAATGCCCACATGCC-3’  5’-TTTGGCAATGGTGGTACAGC-3’  5’-CAGACATCGACCTCATGGTG-3’ |
|  | Reverse | 5’-AGGCATAGTGGACCTTGGTG-3’ |
| 18S rRNA | Forward | 5’-CGGCTACCACATCCAAGGAA-3’ |
|  | Reverse | 5’-GCTGGAATTACCGCGGCT-3’ |

MHC, myosin heavy chain; Fndc5, fibronectin type III domain-containing 5; IGF-1, insulin-like growth factor-1; FGF2, fibroblast growth factor 2; TGF-β, transforming growth factor-β; IL-6, interleukin-6; OLFM1, Olfactomedin 1.
