# Supplementary figures and images for: Effects of elastase-induced emphysema on muscle and bone in mice
Source: PLoS One. 2023 Jun 23;18(6):e0287541. doi: 10.1371/journal.pone.0287541 (PMC10289373; doi:10.1371/journal.pone.0287541)

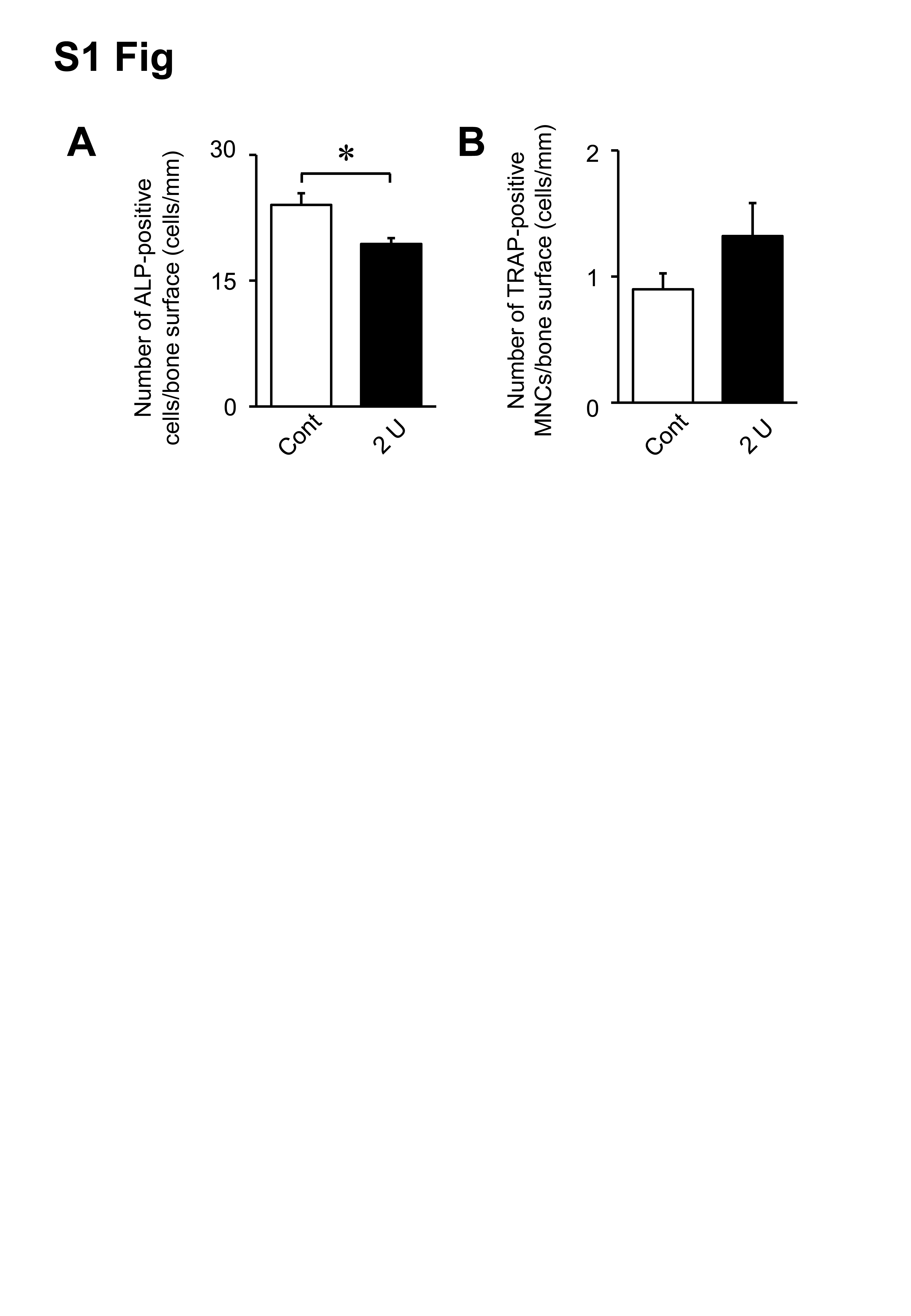

Supplement: S1 Fig — (A) Immunohistochemistry for ALP was performed on the femurs of mice 8 weeks after the intratracheal administration of saline or 2 U PPE. The number of ALP-positive cells on the bone surface of femoral trabecular bone was counted. (B) TRAP staining was performed on the femurs of mice 8 weeks after the intratracheal administration of saline or 2 U PPE. The number of TRAP-positive MNCs on the bone surface of femoral trabecular bone was counted. Data represent the mean ± SEM. n = 8 (Control) and 5 (2 U) mice. *p <0.05. (TIF) [file pone.0287541.s002.tif]
